# Chlamydial Pre-Infection Protects from Subsequent Herpes Simplex Virus-2 Challenge in a Murine Vaginal Super-Infection Model

**DOI:** 10.1371/journal.pone.0146186

**Published:** 2016-01-04

**Authors:** Jessica Slade, Jennifer V. Hall, Jennifer Kintner, Robert V. Schoborg

**Affiliations:** Department of Biomedical Sciences, Center for Inflammation, Infectious Disease and Immunity, James H. Quillen College of Medicine, East Tennessee State University, Johnson City, Tennessee, United States of America; University of the Pacific, UNITED STATES

## Abstract

*Chlamydia trachomatis* and Herpes Simplex Virus-2 (HSV-2) genital tract co-infections have been reported in humans and studied *in vitro* but the clinical consequences are unknown. Limited epidemiologic evidence suggests that these co-infections could be more severe than single infections of either pathogen, but the host-pathogen interactions during co-infection remain uncharacterized. To determine whether disease progression and/or pathogen shedding differs between singly-infected and super-infected animals, we developed an *in vivo* super-infection model in which female BALB/c mice were vaginally infected with *Chlamydia muridarum* (Cm) followed later by HSV-2. Pre-infection with *Chlamydia* 3 or 9 days prior to HSV-2 super-infection conferred significant protection from HSV-2-induced neurologic disease and significantly reduced viral recovery compared to HSV-2 singly-infected controls. Neither protection from mortality nor reduced viral recovery were observed when mice were i) super-infected with HSV-2 on day 27 post Cm; ii) infected with UV-irradiated Cm and super-infected with HSV-2; or iii) azithromycin-treated prior to HSV-2 super-infection. Therefore, protection from HSV-2-induced disease requires active infection with viable chlamydiae and is not observed after chlamydial shedding ceases, either naturally or due to antibiotic treatment. Thus, *Chlamydia*-induced protection is transient and requires the continued presence of chlamydiae or their components. These data demonstrate that chlamydial pre-infection can alter progression of subsequent HSV-2 infection, with implications for HSV-2 transmission from co-infected humans.

## Introduction

Herpes Simplex Virus Type-2 and *Chlamydia trachomatis* are two of the most common sexually transmitted pathogens in the world. More than 500 million people aged 15–49, or 16% of the population in this age range, are living with HSV-2 [1]. *C*. *trachomatis* is the most common sexually transmitted bacterial pathogen, causing over 100 million genital tract infections per year worldwide [2]. In the United States alone, the CDC estimated that there were 110 million new and existing sexually transmitted infections (STIs) in 2008, with nearly 50% occurring in young people aged 15–24 [3]. Of these STIs, there are a combined 25 million Herpes Simplex Virus-2 (HSV-2) and *C*. *trachomatis* cases each year [3].

HSV-2 is an enveloped DNA virus of the family, *Herpesviridae*, and is the primary cause of genital herpes infection. HSV-2 infection usually occurs on the mucous membranes and skin surrounding the genitals causing characteristic, often painful, lesions [1]. After primary infection, HSV-2 establishes a latent, life-long infection in the neurons of the sacral ganglia [4]. Reactivations from latency are often brief, with some lasting less than 12 hours, but occur as many as 18 times per year without the use of suppressive therapy [5]. In humans, most HSV-2 infections and reactivations are asymptomatic or clinically mild, though serious diseases, like keratitis and meningitis, can result [4, 5]. More women than men are HSV-2 infected and transmission of HSV-2 during childbirth can cause complications in neonates resulting in brain damage or death [1]. Treatment of HSV-2 with antiviral drugs such as valacyclovir does not cure an infection but can reduce viral shedding [6], which also decreases the transmission risk [7].

The chlamydiae are obligate intracellular, bacterial pathogens. *C*. *trachomatis* serovars D-K are the primary cause of chlamydial genital tract infections in the US but are asymptomatic in 50–70% of infected individuals [8]. *C*. *trachomatis* infections can be treated with antibiotics such as azithromycin. However, the asymptomatic nature of these infections often leads to chronic inflammation, such as urethritis or proctitis in men and cervicitis in women, because infections go untreated. Women can experience severe outcomes as the chlamydiae ascend the genital tract, including pelvic inflammatory disease (PID), infertility and ectopic pregnancy [8, 9].

Genital tract co-infections with HSV-2 and *C*. *trachomatis* have been reported in infected men [10] and women [11–14]. One epidemiologic study suggests that women who are positive for both HSV-2 and *C*. *trachomatis* may experience more severe outcomes, such as endometritis and salpingitis, than are typically experienced during single infections with each pathogen [15]. Fertility related complications, such as spontaneous abortion, have also been reported in co-infected women [16]. Unfortunately, most studies evaluating *Chlamydia* and HSV co-infections have been designed to investigate the prevalence of STIs within a population rather than to specifically examine infection outcomes. These epidemiologic studies rely heavily on serological data, making it difficult to determine whether or not both pathogens were present simultaneously in the genital tract at any given time [11, 13, 14]. Though published data suggest that co-infection might alter disease outcome, the exact pathogen-pathogen and/or host-pathogen interactions that occur during *Chlamydia* and HSV-2 co-infections, and the resulting clinical consequences, remain essentially unexplored. Thus, we chose to begin addressing these questions by performing super-infections in an experimentally-tractable animal model to avoid the limitations of serologic human retrospective studies.

There are many reported instances of one pathogen altering the disease progression of another *in vivo*, typically to the detriment of the host. For example, Influenza A virus normally causes self-limiting upper respiratory tract infections. However, influenza infection can alter respiratory tract physiology in ways that pre-dispose the host to develop life-threatening secondary bacterial pneumonia [17]. Moreover, progression of Human Immunodeficiency Virus (HIV) infection is exacerbated by co-infection with either HSV-2 or *C*. *trachomatis*. During HSV-2 and HIV co-infection, HSV-2 not only increases the recruitment of DC-sign expressing dendritic cells to the genital mucosa, increasing HIV replication, but also increases the transmission rates of both viruses [11, 18–20]. HIV shedding also significantly increases during co-infection with *C*. *trachomatis*, especially in instances of cervicitis [21]. Women with HIV are at higher risk for acquiring *C*. *trachomatis* infection and are also more likely to experience PID than HIV negative women [22, 23]. Therefore, it seems likely that disease severity, progression, and/or chlamydial/viral transmission of HSV-2 and *C*. *trachomatis* co-infected individuals would be altered from that observed in single infections of either pathogen.

*Chlamydia* share a unique biphasic developmental cycle in which they enter the host as the infectious, extracellular form called elementary bodies (EB). The EB, upon entering the host cell begin to differentiate into the non-infectious, replicative form called reticulate bodies (RB). The RB grow and divide within a membrane bound vacuole termed an inclusion. After a few rounds of division, the RB redifferentiate into EB which leave the host cell and infect neighboring cells [24]. Through the use of an *in vitro* super-infection model, we previously demonstrated that HSV super-infection of *Chlamydia*-infected cells causes the chlamydiae to deviate from the normal developmental cycle [25, 26]. This decreases EB production from super-infected cultures compared to cells that are singly-infected with *C*. *trachomatis* or *Chlamydia muridarum*. To further dissect the interactions between host and pathogen that occur during *Chlamydia* and HSV-2 co-infections, we developed an *in vivo C*. *muridarum* and HSV-2 murine genital tract super-infection model. *C*. *muridarum* is widely used as an *in vivo* infection model because mice are susceptible to ascending infection with *C*. *muridarum* and comparable pathology to human *C*. *trachomatis* genital tract infections is observed [27–30]. A similar murine genital infection model is also used to study HSV-2 pathogenesis *in vivo*, although the outcome of disease is significantly more severe than that usually seen in humans [31–33].

HSV-2 and *C*. *trachomatis* co-infections have been reported in humans but the clinical consequences and the host-pathogen interactions that occur during these co-infections remain largely unknown. We previously observed that chlamydial development is altered during *C*. *trachomatis and* HSV super-infection *in vitro*. Based upon these observations, we hypothesized that *in vivo*, disease progression and/or pathogen shedding in *C*. *muridarum and* HSV-2 super-infected animals would differ from that observed in animals singly-infected with either pathogen. Here, we developed a novel *C*. *muridarum* and HSV-2 murine genital tract super-infection model to demonstrate that chlamydial infection reduces both viral recovery and neuroinvasive disease subsequent to a vaginal HSV-2 challenge.

## Results

### Disease progression and pathogen shedding differ between singly-infected and super-infected animals

**Fig 1:**
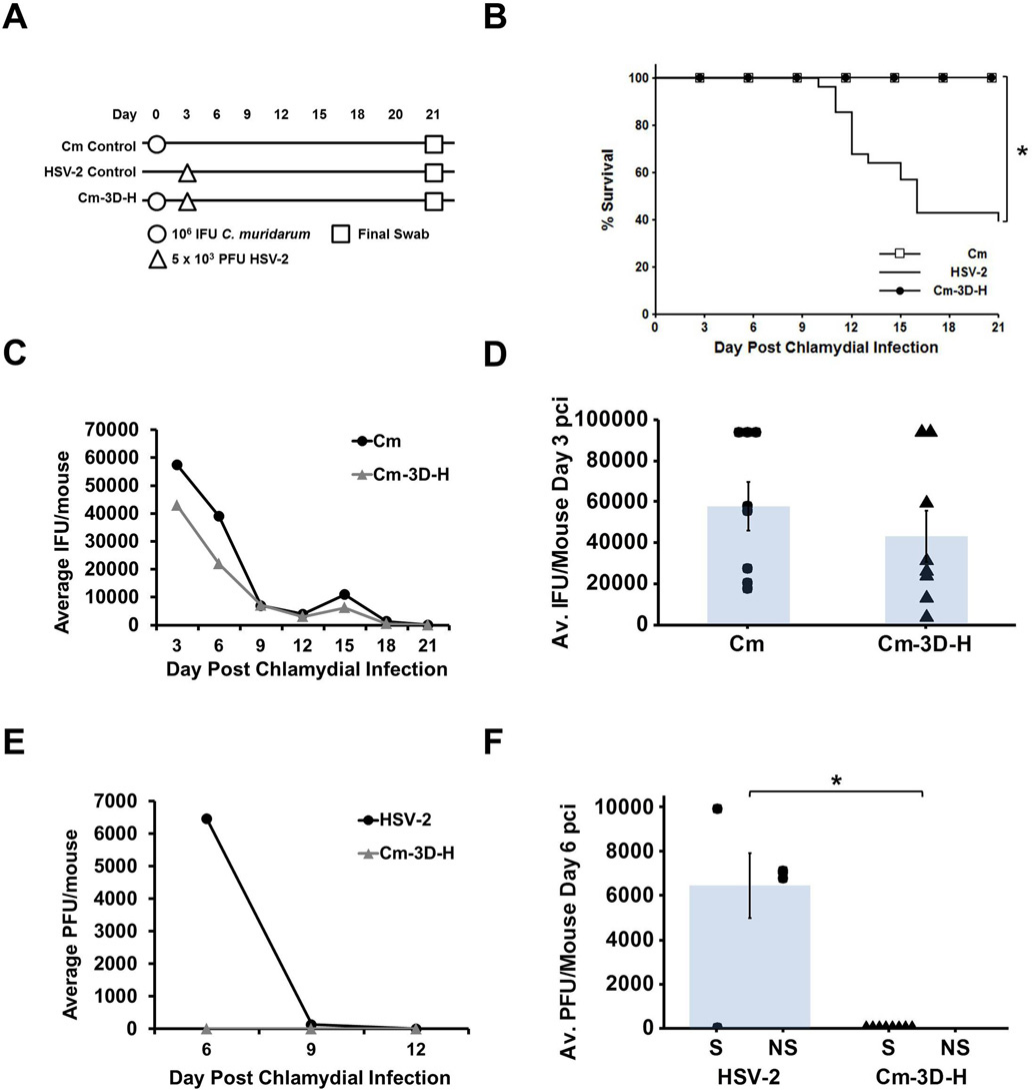
Disease progression and pathogen shedding differ between singly-infected and super-infected animals. (A) Experimental design for *C*. *muridarum* and HSV-2 genital tract super-infection. Female BALB/c mice were singly-infected with 10^6^ IFU Cm on day 0 post chlamydial infection (pci) or 5 x 10^3^ PFU HSV-2 on day 3 pci. Super-infected mice were vaginally infected with Cm on day 0 pci followed by HSV-2 on day 3 pci. Vaginal swabbing was performed on all mice every 3 days until day 21 pci. (B) Survival observed in Cm singly-infected mice (Cm), HSV-2 singly-infected mice (HSV-2) and super-infected mice (Cm-3D-H). Morbidity and mortality resulting from HSV-2 was monitored daily and the percent survival between experimental groups was compared using the log rank statistic. Significant (p<0.05) difference between singly-infected controls or between the HSV-2 singly-infected control and super-infected groups is indicated by an asterisk (*). The survival curve depicted is the combined data from four separate experiments with n = 32 for Cm, n = 44 for HSV-2, and n = 40 for Cm-3D-H. (C) Chlamydial shedding was determined by chlamydial titer assay and is reported as average IFU/mouse +/- SEM. (D) Average chlamydial shedding at day 3 pci (indicated by bars) and individual mouse chlamydial shedding (segregated according to survival status) are shown; n = 8 for both Cm (circles) and Cm-3D-H (triangles). (E) HSV-2 recovery was determined by plaque assay and is reported as average PFU/mouse +/- SEM. (F) Average HSV-2 shedding at day 6 pci (indicated by bars) and individual mouse HSV-2 recovery (segregated according to survival status) is shown; n = 6 for HSV-2 (circles) and n = 8 for Cm-3D-H (triangles). Survivors and non-survivors are indicated by S and NS, respectively. The data in panels C-F are representative of 4 independent experiments. Differences in pathogen shedding/recovery between groups were determined with the paired Student’s t-test with p<0.05 considered significant, as indicated by an asterisk (*).

Through the use of our *in vitro* super-infection model, we previously observed that chlamydial development is hindered by HSV-2 super-infection [25]. To begin characterizing the disease progression of *Chlamydia* and HSV-2 within the co-infected host, mice were vaginally infected with 10^6^ IFU *C*. *muridarum* (Cm) on day 0 and then super-infected with 5 x 10^3^ PFU HSV-2 on day 3 post chlamydial infection (pci). Vaginal swabbing was performed every 3 days until day 21 pci (Fig 1A); chlamydial shedding was determined by chlamydial titer assay and viral recovery was determined by plaque assay for HSV-2 (Fig 1C and 1D; Fig 1E and 1F). HSV-2 infection causes neurological disease in mice, which first manifests as hind limb paralysis and results in death [32], therefore mice were monitored daily for HSV-2-induced morbidity and mortality. Mice exhibiting hind limb paralysis were euthanized and incorporated into the survival data (Fig 1B) as being susceptible to HSV-2.

By day 21 pci, Cm singly-infected mice exhibited 100% survival whereas HSV-2 singly-infected mice exhibited only 40% survival, which was both expected and significantly different (p<0.05). Interestingly, when mice were infected first with *C*. *muridarum* on day 0 and then super-infected with HSV-2 on day 3 pci (Cm-3D-H), 100% of the mice survived. These data indicate that chlamydial pre-infection offers significant protection from HSV-2-induced neuroinvasive disease (p<0.05; Fig 1B).

Chlamydial shedding between the Cm and Cm-3D-H groups was similar throughout the course of the infection, including peak shedding on day 3 pci (Fig 1C and 1D). Viral recovery, however, was significantly different between HSV-2 singly-infected animals and the super-infected group at day 6 pci; in fact, no detectable virus was recovered from super-infected animals (p<0.05; Fig 1E and 1F).

A similar protective effect was observed when mice were simultaneously infected with a combined inoculum of 10^6^ IFU Cm and 5 x 10^3^ HSV-2 on day 0. Simultaneously-infected mice (Sim) exhibited 90% survival compared to HSV-2 singly-infected controls, which showed 30% survival (p<0.05; S1A Fig). Viral recovery was significantly reduced in simultaneously-infected animals compared to HSV-2 singly-infected controls (p<0.05; S1B and S1C Fig), as was observed when Cm is inoculated 3 days prior to HSV-2 infection (Fig 1E and 1F).

### Chlamydial pre-infection protects from challenge with a 100% lethal dose of HSV-2

**Fig 2:**
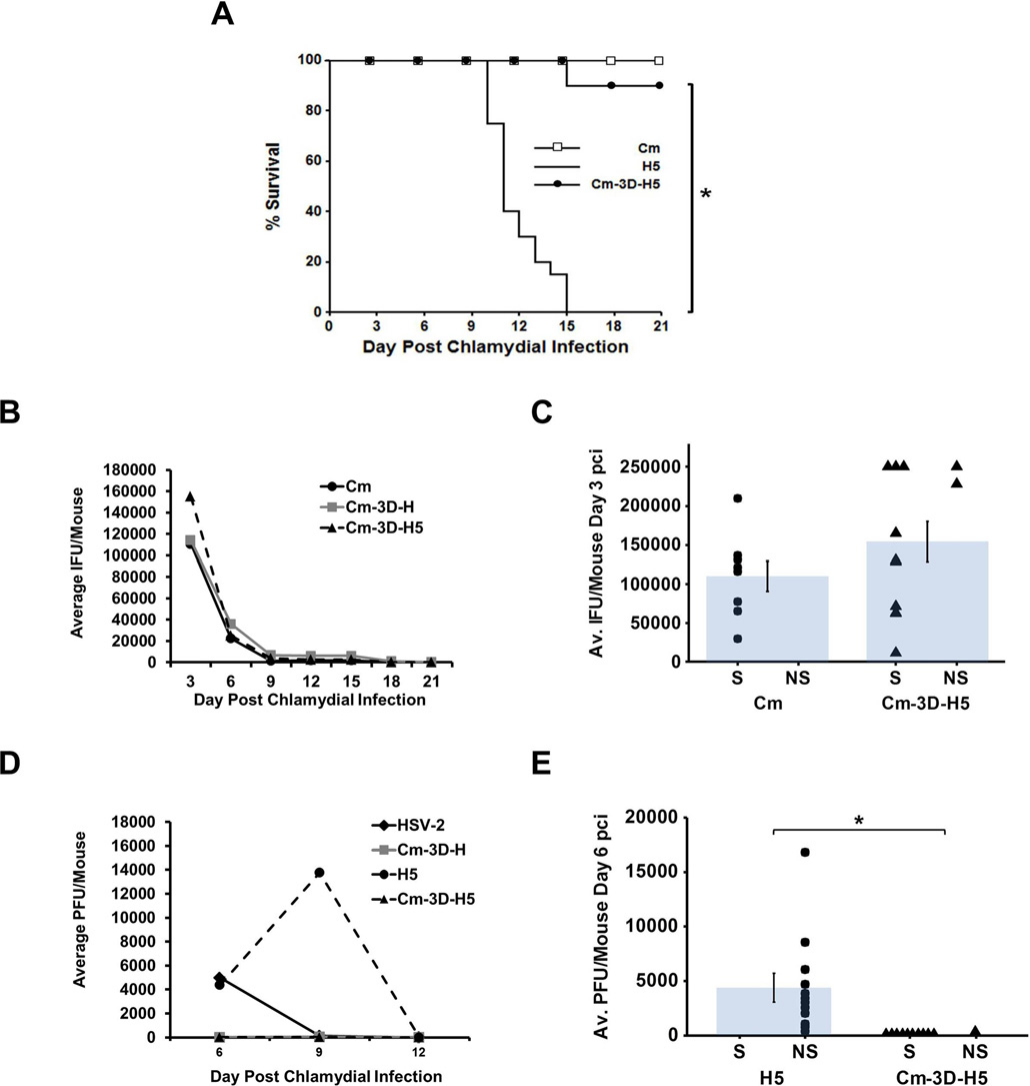
Chlamydial pre-infection protects from challenge with a 100% lethal dose of HSV-2. (A) Female BALB/c mice were singly-infected with either 10^5^ PFU HSV-2 (H5) or with 10^6^ IFU *C*. *muridarum* (Cm). Super-infected mice were infected with Cm on day 0 pci and the high inoculum of HSV-2 on day 3 pci (Cm-3D-H5). Morbidity and mortality resulting from HSV-2 was monitored daily and the percent survival between experimental groups was compared using the log rank statistic. Significant (p<0.05) difference between HSV-2 singly-infected controls and super-infected group is indicated by an asterisk (*). The survival curve depicts data combined from 2 separate experiments with n = 16 for Cm and n = 20 for both the H5 and Cm-3D-H5 groups. (B) Chlamydial shedding was determined by chlamydial titer assay and is reported as average IFU/mouse +/- SEM. (C) Average chlamydial shedding at day 3 pci (indicated by bars) and individual mouse chlamydial shedding (segregated according to survival status) are shown; n = 8 for Cm (circles) and n = 12 for Cm-3D-H (triangles). (D) HSV-2 recovery was determined by plaque assay and is reported as average PFU/mouse +/- SEM. (E) Average HSV-2 recovery at day 6 pci (indicated by bars) and individual mouse HSV-2 recovery (segregated according to survival status) are shown; n = 12 for both HSV-2 (circles) and Cm-3D-H (triangles). (C and E) Survivor and non-survivor mice are indicated by S and NS, respectively. The data in panels C-F are representative of 2 independent experiments. Differences in pathogen shedding/recovery between groups were determined with the paired Student’s t-test with p<0.05 considered significant, as indicated by an asterisk (*).

To ascertain whether or not the protective effect elicited by chlamydial pre-infection was HSV-2 dose-dependent, we also super-infected using a higher inoculum of 10^5^ PFU HSV-2. This dose was 100% lethal in HSV-2 singly-infected mice (H5) by day 15 pci. In contrast, super-infected mice (Cm-3D-H5) exhibited 90% survival by day 21 pci (p<0.005; Fig 2A). Again, chlamydial shedding was not different between Cm singly-infected mice and the Cm-3D-H5 groups, nor was chlamydial shedding different between mice super-infected with our standard HSV-2 inoculum of 5 x 10^3^ (Cm-3D-H) and the high inoculum of HSV-2 (Cm-3D-H5) at peak recovery (p = 0.03; Fig 2B and 2C). As before, viral recovery was significantly reduced in Cm-3D-H5 super-infected mice at day 6 (p<0.05; Fig 2D and 2E) and at day 9 pci compared to H5 singly-infected mice. At day 6pci, HSV-2 recovery for Cm-3D-H5 survivors was 0 PFU, but recovery in the non-survivors averaged 116 PFU/mouse, which is not visible on the scale used (Fig 2E). Although viral recovery peaked later than in the previous experiments (Fig 1D), these data are consistent with the peak shedding ranges previously observed in HSV vaginally-infected mice given the 3 day sampling interval [31, 34–36].

### Protection elicited by chlamydial pre-infection is time-dependent

**Fig 3:**
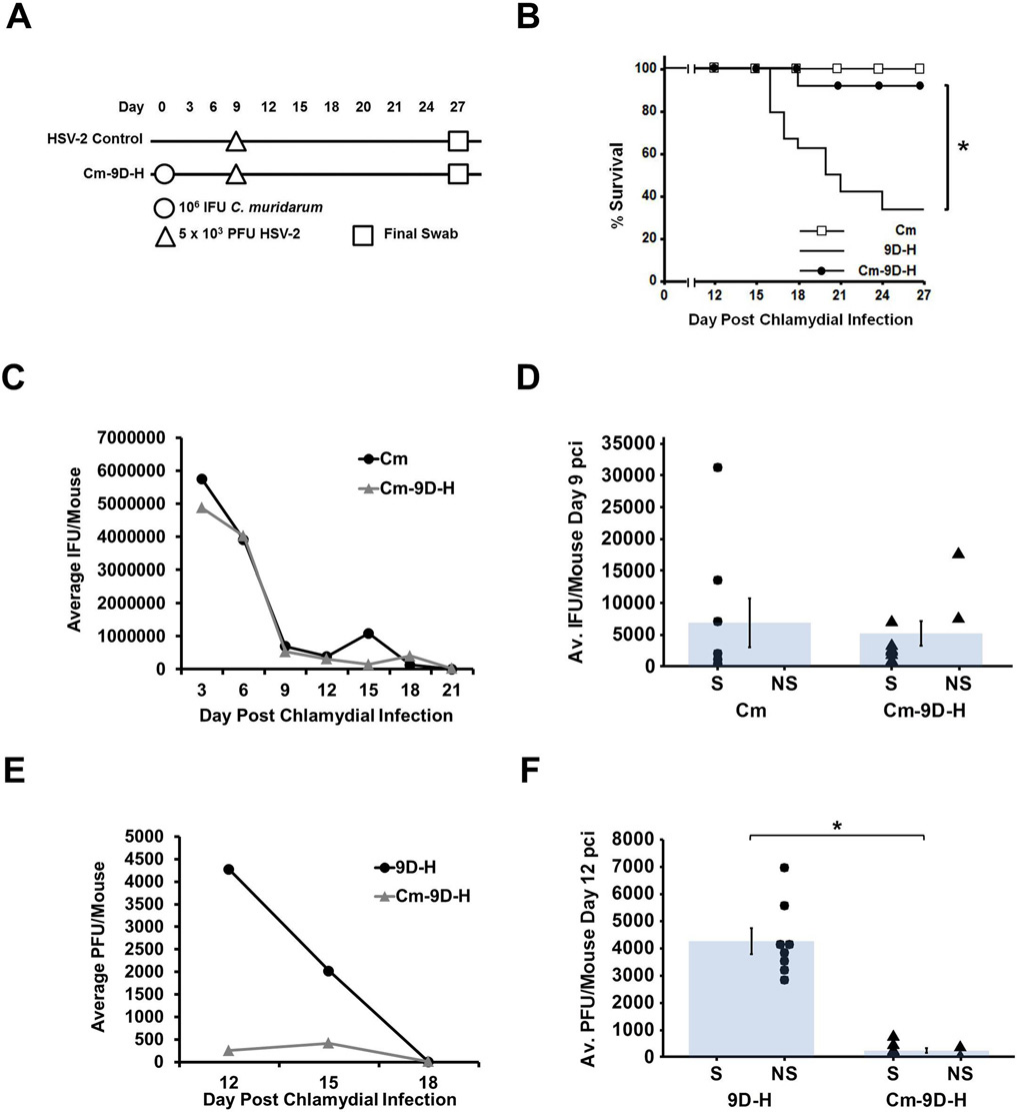
Protection elicited by chlamydial pre-infection lasts until at least day 9 pci. (A) Mice were super-infected with 10^6^ IFU *C*. *muridarum* on day 0 pci then with 5 x 10^3^ PFU HSV-2 on day 9 pci (Cm-9D-H) and swabbed every three days until day 27 pci. (B) Morbidity and mortality resulting from HSV-2 was monitored daily and the percent survival between experimental groups was compared using the log rank statistic. Significant (p<0.05) difference from Cm singly-infected control is indicated by an asterisk (*). The survival curve includes data combined from 3 separate experiments with n = 24 for each group. (C) Chlamydial shedding was determined by chlamydial titer assay and is reported as average IFU/mouse +/- SEM. (D) Average chlamydial shedding at day 9 pci (indicated by bars) and individual mouse chlamydial shedding (segregated according to survival status) are shown; n = 8 for both Cm (circles) and Cm-9D-H (triangles). (E) HSV-2 recovery was determined by plaque assay and is reported as average PFU/mouse +/- SEM. (F) Average HSV-2 recovery at day 12 pci (indicated by bars) and individual mouse HSV-2 recovery (segregated according to survival status) are shown; n = 8 for both HSV-2 (circles) and Cm-3D-H (triangles). Survivors and non-survivors are indicated by S and NS, respectively. The data in panels C-F are representative of 3 independent experiments. Differences in pathogen shedding/recovery between groups were determined with the paired Student’s t-test with p<0.05 considered significant, as indicated by an asterisk (*).

**Fig 4:**
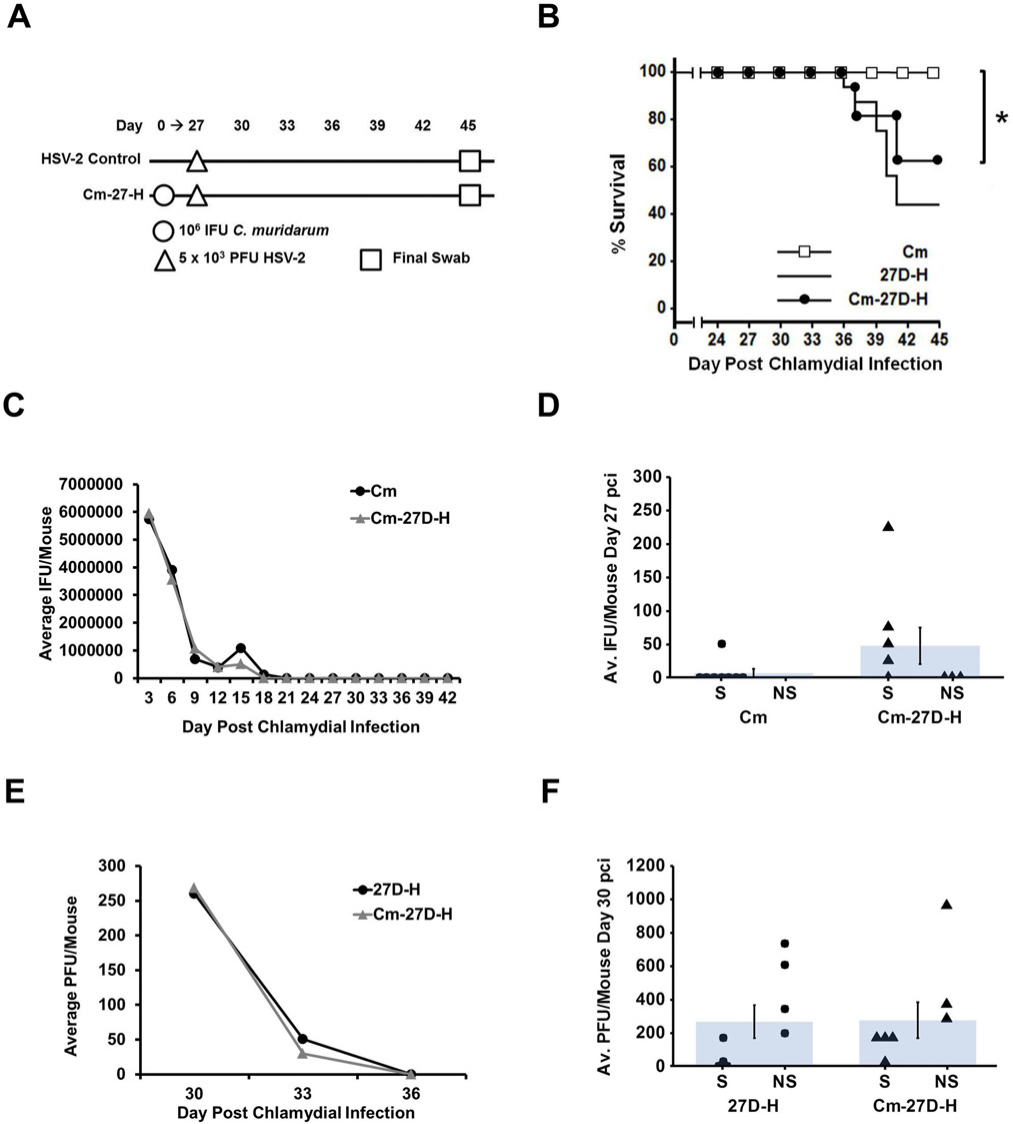
Protection elicited by chlamydial pre-infection is lost by day 27 pci. (A) Mice were super-infected with 10^6^ IFU *C*. *muridarum* on day 0 pci then with 5 x 10^3^ HSV-2 on day 27 pci (Cm-27D-H) and swabbed every 3 days until day 45 pci. (B) Morbidity and mortality resulting from HSV-2 was monitored daily and the percent survival between experimental groups was compared using the log rank statistic. Significant (p<0.05) difference from HSV-2 singly-infected control is indicated by an asterisk (*).The survival curve contains data combined from 2 separate experiments with n = 16 for each group. (C) Chlamydial shedding was determined by chlamydial titer assay and is reported as average IFU/mouse +/- SEM. (D) Average chlamydial shedding at day 27 pci (indicated by bars) and individual mouse chlamydial shedding (segregated according to survival status) are shown; n = 8 for both Cm (circles) and Cm-9D-H (triangles). (E) HSV-2 recovery was determined by plaque assay and is reported as average PFU/mouse +/- SEM. (F) Average HSV-2 recovery at day 30 pci (indicated by bars) and individual mouse HSV-2 shedding (segregated according to survival status) are shown; n = 8 for both HSV-2 (circles) and Cm-3D-H (triangles). Survivors and non-survivors are indicated by S and NS, respectively. The data in panels C-F are representative of 2 independent experiments. Differences in pathogen shedding/recovery between groups were determined with the paired Student’s t-test with p<0.05 considered significant, as indicated by an asterisk (*).

Because we observed protection from HSV-2-induced disease in mice pre-infected with Cm 3 days prior to HSV-2 super-infection, we wanted to determine whether this protection was long-lasting. Mice were infected first with Cm on day 0 as before, but then super-infected with HSV-2 at either day 9 (Fig 3A) or day 27 pci (Fig 4A). We chose these time points by examining a typical Cm shedding curve. By day 9 pci, chlamydial shedding, while still detectable, has declined significantly from peak shedding at day 3 or day 6 pci. By day 27 pci, chlamydial shedding is no longer detectable [37]. In mice super-infected with HSV-2 on day 9 pci, protection was still observed and the super-infected mice (Cm-9D-H) exhibited significantly higher survival compared to the HSV-2 singly-infected mice (p<0.005), although survival in the Cm-9D-H group dropped slightly to 90% (Fig 3B). As before, chlamydial shedding did not differ between Cm and Cm-9D-H groups (Fig 3C and 3D). In contrast, viral recovery was significantly reduced in the Cm-9D-H group compared to HSV-2 singly-infected controls, though some virus was detectable on day 12 pci (p<0.05; Fig 3E and 3F). By day 27 pci, infectious chlamydiae were not detectable in vaginal swabs from Cm singly-infected animals, although a small but not-significantly different number of chlamydiae were detected in the Cm-27D-H samples (Fig 4C and 4D). Survival in the Cm-27D-H group dropped to 60%, which was statistically indistinguishable from that observed in HSV-2 singly-infected mice (40%, Fig 4B). Viral recovery was no longer significantly different between the Cm-27D-H group and the HSV-2 singly infected controls on day 30 pci (Fig 4E and 4F). Thus, the protective effect elicited by chlamydial pre-infection appears to be transient and may require the presence of viable chlamydiae in the genital tract at the time of HSV-2 challenge.

The observation that *Chlamydia*-induced protection is time-dependent was confirmed when the super-infection was performed in the reverse order. Mice were infected with HSV-2 on day 0 then super-infected with Cm on day 3 post HSV-2 infection (phi) (H-3D-Cm). As controls, mice were singly-infected with HSV-2 on day 0 or Cm on day 3 phi (H-D0, 3D-Cm) (S2A Fig). The H-3D-Cm super-infected mice exhibited an intermediate level of protection from HSV-2-induced disease, as their survival was significantly different from both control groups (p<0.05; S2B Fig). Viral recovery was not significantly different at any sampling time (S2E Fig; p = 0.22, S2F Fig). However, chlamydial shedding in the super-infected group was significantly lower than the 3D-Cm group at day 6 phi (p<0.005) returning to normal levels by day 9 phi (S2C and S2D Fig), suggesting that established HSV-2 vaginal infection alters subsequent *C*. *muridarum* infection kinetics. Overall, these data indicate that: i) the protective effect of Cm is transient; and ii) Cm can provide at least partial protection, even when inoculated after HSV-2 infection.

### Active chlamydial infection is required to protect from HSV-2-induced disease

**Fig 5:**
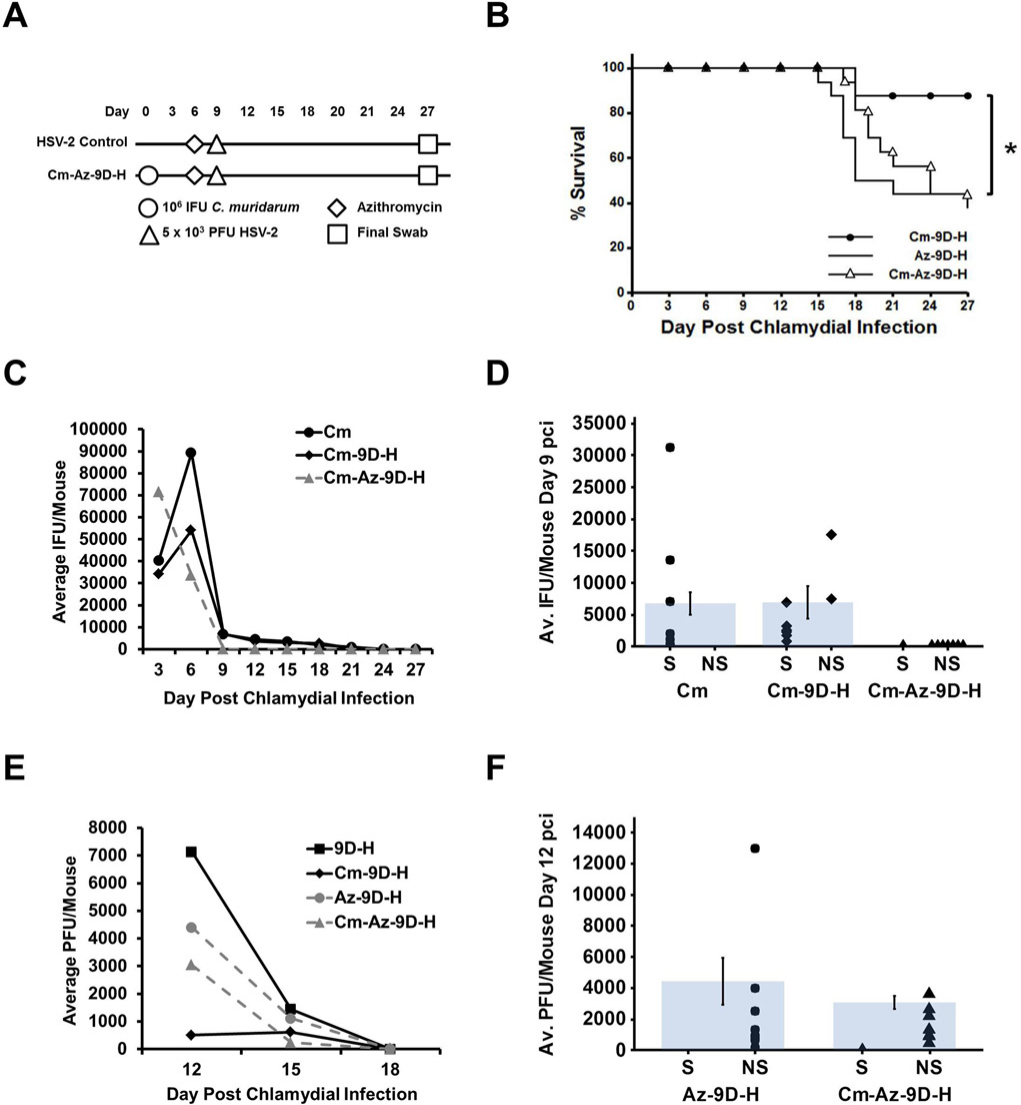
Active chlamydial infection is required to protect from HSV-2-induced disease. (A) Mice were infected with 10^6^ IFU *C*. *muridarum* on day 0 pci and then treated with 200 mg/kg azithromycin (Az) via oral gavage on day 6 pci to cure the chlamydial infection. On day 9 pci, the mice were super-infected with 5 x 10^3^ PFU HSV-2. As a control, mice were treated with Az on day 6 pci then singly-infected with HSV-2 on day 9 pci. Pathogen shedding was determined from swab samples performed every 3 days until day 27 pci. (B) Morbidity and mortality resulting from HSV-2 was monitored daily and the percent survival between experimental groups was compared using the log rank statistic. Significant (p<0.05) difference from Cm singly-infected control is indicated by an asterisk (*). The survival curve depicts data combined from 2 separate experiments with n = 16 for each group. (C) Chlamydial shedding was determined by chlamydial titer assay and is reported as average IFU/mouse +/- SEM. (D) Average chlamydial shedding at day 9 pci (indicated by bars) and individual mouse chlamydial shedding (segregated according to survival status) are shown; n = 8 for Cm (circles), Cm-9D-H (diamonds) and Cm-Az-9D-H (triangles). (E) HSV-2 recovery was determined by plaque assay and is reported as average PFU/mouse +/- SEM. (F) Average HSV-2 recovery at day 12 pci (indicated by bars) and individual mouse HSV-2 shedding (segregated according to survival status) are shown; n = 8 for both Az-9D-H (circles) and Cm-Az-9D-H (triangles). Survivors and non-survivors are indicated by S and NS, respectively. Differences in pathogen shedding/recovery between groups in panels C-F were determined with the paired Student’s t-test with p<0.05 considered significant and are representative of 2 independent experiments.

The loss of protection observed in mice super-infected with Cm on day 0 and HSV-2 on day 27pci coincided with the loss of detectable chlamydiae in genital tract swab samples at the time of HSV-2 infection. To determine whether cessation of chlamydial shedding from the genital tract results in a loss of protection from HSV-2-induced disease, mice were infected with Cm then treated with 200 mg/kg azithromycin (Az) by oral gavage on day 6 pci to cure the chlamydial infection (Fig 5A) [37]. Cm titer assays confirmed the success of the treatment, as Cm shedding reached 0 IFU by day 9 (Fig 5C and 5D). At day 9, mice were super-infected with 5 x 10^3^ PFU HSV-2. Az-treated mice that were HSV-2-infected on day 9 (Az-9D-H) exhibited similar mortality to untreated HSV-2 singly-infected controls, demonstrating that Az treatment does not affect HSV-2 infection outcome (Fig 5B). Compared to Cm-9D-H untreated controls, Az-treated, HSV-2 super-infected animals (Cm-Az-9D-H) exhibited significantly higher mortality (p<0.05). In fact, Cm-Az-9D-H mortality was indistinguishable from that of Az-9D-H controls (p = 0.44; Fig 5B). Viral recovery in the Cm-Az-9D-H group was not significantly different from that observed in the Az-9D-H group (Fig 5E and 5F), but was different compared to the untreated, HSV-2 super-infected controls (Cm-9D-H, p<0.05; Fig 5E). These data suggest that actively-replicating chlamydiae must be present in the genital tract at the time of HSV-2 infection to elicit the protective effect.

### Protection from HSV-2 induced disease requires viable chlamydiae

**Fig 6:**
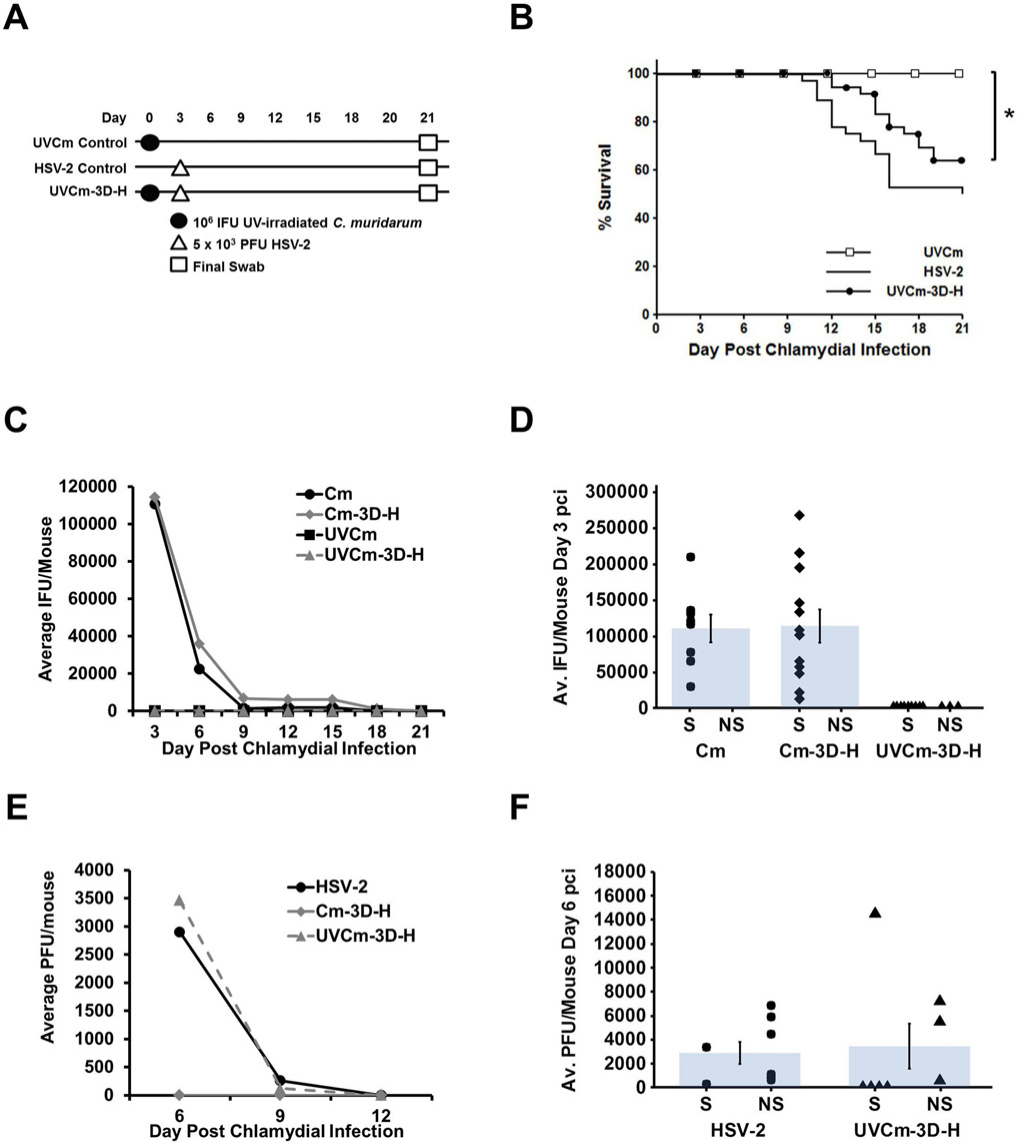
Protection from HSV-2-induced disease requires viable chlamydiae. (A) Mice were super-infected with 10^6^ UV-irradiated Cm (UVCm) then with 5 x 10^3^ PFU HSV-2 on day 3 pci (UVCm-3D-H). As controls, mice were singly-infected with UV-irradiated Cm (UVCm) on day 0 or with HSV-2 on day 3 pci (HSV-2). Vaginal swabbing was performed every 3 days until day 21 pci. (B) Morbidity and mortality resulting from HSV-2 was monitored daily and the percent survival between experimental groups was compared using the log rank statistic. Significant (p<0.05) difference from Cm singly-infected control is indicated by an asterisk (*). The survival curve depicts data combined from 3 separate experiments with n = 24 for UVCm and n = 36 for both the HSV-2 and UVCm-3D-H groups. Note that 100% survival was also observed in mock, Cm, and super-infected Cm-3D-H control groups that were included within this experiment (not shown). (C) Chlamydial shedding was determined by chlamydial titer assay and is reported as average IFU/mouse +/- SEM. (D) Average chlamydial shedding at day 3 pci (indicated by bars) and individual mouse chlamydial shedding (segregated according to survival status) are shown; n = 8 for Cm (circles), and n = 12 for both Cm-3D-H (diamonds) and UVCm-3D-H (triangles). (E) HSV-2 recovery was determined by plaque assay and is reported as average PFU/mouse +/- SEM. (F) Average HSV-2 recovery at day 6 pci (indicated by bars) and individual mouse HSV-2 recovery (segregated according to survival status) are shown; n = 8 for HSV-2 (circles) and n = 12 for UVCm-3D-H (triangles). Survivors and non-survivors are indicated by S and NS, respectively. Differences in pathogen shedding/recovery between groups in panels C-F were determined with the paired Student’s t-test with p<0.05 considered significant and are representative of 3 independent experiments.

To confirm the prediction that actively-replicating chlamydiae are required for protection from HSV-2 disease, our standard inoculum of Cm was replaced with an identical number of UV-irradiated, replication-incompetent organisms on day 0 (UVCm). This was followed by our standard inoculum of 5 x 10^3^ PFU HSV-2 on day 3 (Fig 6A). We included mock, Cm singly-infected mice (Cm), and mice super-infected with viable chlamydiae and HSV-2 (Cm-3D-H), as additional control groups within this experiment (Fig 6C–6F). However, the survival data from the Cm and Cm-3D-H groups is not included on the survival plot (Fig 6B) because they were indistinguishable from that observed in the UVCm group (100% survival) and simply confirm the results presented in Fig 1B. Compared to UVCm singly infected animals, the HSV-2 super-infected group experienced significantly less survival (UVCm-3D-H, 60% survival; p<0.005) and was not significantly different from HSV-2 singly-infected controls (which exhibited 50% survival; Fig 6B). Chlamydial titer assays confirmed that the UVCm inoculum produced no detectable chlamydial progeny (Fig 6C and 6D) and that UVCm-3D-H mice shed no detectable IFU compared to the Cm and Cm-3D-H groups on day 3 pci (which produced 6.8 x 10^3^ and 7 x 10^3^ IFU, respectively; Fig 6D). No significant reduction in viral recovery was observed in the UVCm super-infected group, even at peak recovery at day 6 pci (Fig 6E and 6F). These data indicate that chlamydial pre-infection only protects from HSV-2-induced mortality while the chlamydiae are actively-replicating and viable chlamydiae are present.

## Discussion

There are many documented instances of one pathogen altering the disease progression of another *in vivo*. Such co-infections tend to result in a more severe outcome for the host [17, 18, 21]. However, some co-infections can benefit the host. Historically, neurosyphilis caused by the sexually transmitted pathogen, *Treponema pallidum*, was successfully treated by the fever resulting from the inoculation of patients with *Plasmodium vivax*, the causative agent for malaria [38, 39]. There are several examples of mouse models in which one pathogen protects from a secondary infection. Pre-infection with intracellular protozoan *Toxoplasma gondii* 5 days prior to infection with *Leishmania major* protected against *L*. *major* footpad lesion development [40]. Prior infection with either *Listeria monocytogenes* or *Mycobacterium tuberculosis* can protect from subsequent lethal infection from *Plasmodium yoelii* [41, 42]. In each instance, the establishment of protection against the secondary infection was attributed to the production of pro-inflammatory cytokines by the first pathogen [40–42].

We developed a *Chlamydia muridarum* and HSV-2 super-infection model to further understand the disease progression of *Chlamydia* and HSV-2 STIs within the host genital tract. Surprisingly, this super-infection scenario benefits the host when mice are pre-infected with *Chlamydia* either prior to, simultaneously with, or shortly after challenge with HSV-2, by reducing the frequency of HSV-2 lethal neurologic disease. Chlamydial pre-infection both protects from HSV-2-induced mortality and reduces viral recovery for at least 9 days post-chlamydial infection (pci). The protective effect is lost when chlamydial shedding from the genital tract ceases, either naturally or due to antibiotic treatment. Loss of protection, unsurprisingly, is accompanied by elevated levels of virus recovery. Replacing live chlamydiae with UV-irradiated, replication-incompetent chlamydiae failed to elicit significant protection from HSV-2-induced mortality, indicating that the continued presence of viable chlamydiae (or their components) is required to stimulate the protective effect. Because prior protection is established so early in the course of chlamydia infection (between day 0 and day 9 pci), it seems likely that the protective mechanism requires the activation of specific host innate immune responses rather than adaptive immune responses that happen later in infection [43].

Activation of toll-like receptors (TLRs) is an essential first step in the initiation of the host innate immune response to a variety of pathogens. TLRs are a family of pattern recognition receptors (PRRs) that recognize certain pattern associated molecular patterns (PAMPs) from various types of pathogens—including bacteria, viruses and fungi [44]. During single infections of *Chlamydia*, a number of TLRs are required to clear the infection. Although *Chlamydia muridarum* infection stimulates host cell TLRs 2, 3, 4 and 9 in culture, only TLRs 2 and 3 appear to be required for clearance of genital tract infections in mice [45]. Though the specific chlamydial ligand for TLR2 is unknown, live chlamydiae are required to induce TLR2-related immune responses *in vitro*, as demonstrated by the observation that UV-irradiated chlamydiae fail to elicit the production of mouse macrophage-derived inflammatory mediators [46]. As we also observed a loss of protection from HSV-2-induced disease in our super-infection model when we replaced live chlamydiae with UV-irradiated *C*. *muridarum*, TLR2 may be involved in eliciting protection from subsequent HSV-2 challenge. *In vitro*, HSV-2 appears to induce only limited TLR2 activity and the specific HSV-2 TLR2 ligand is unknown [47]. This low activation could be explained by the ability of HSV-2 to inhibit the expression of TLR2 *in vitro* [48], suggesting that evading TLR2-induced responses is an important survival mechanism for HSV-2. Furthermore, patients with single nucleotide polymorphisms in TLR2 experience increased viral shedding and lesion rate [49], indicating that TLR2 plays an important role in controlling HSV-2 infection in humans. Therefore, chlamydial pre-infection could stimulate such a robust immune response through the activation of TLR2 prior to HSV-2 infection that HSV-2-related disease progression and viral replication are thwarted.

Spread of HSV-2 from the genital mucosa to the ganglia has been observed in mice as early as day 4 pi [50]. At an HSV-2 inoculum comparable to that used in our model, spread to the nervous system was detectable by day 5 pi [31]. Interestingly, we observe an intermediate level of protection when mice were infected first with HSV-2 and challenged 3 days later with *C*. *muridarum* (S2 Fig). Although neuronal entry was not accessed in our experiments, these data collectively suggest at least two, non-exclusive, possibilities. First, the chlamydia-induced, protective response produces a very rapid reduction in HSV-2 genital tract titer, which is sufficient to restrict subsequent neuronal entry and provide protection, even when the chlamydiae are inoculated only a short time before nervous system entry would normally occur. Second, and perhaps more interesting, chlamydial infection may limit HSV-2 neuropathology at some step after the virus enters the nervous system. Notably, neither possibility rules out a role for host TLR2 in chlamydia-induced protection. Thus, defining the role of TLR2 and examining the kinetics of HSV spread into the nervous system in super-infected mice will be a critical part of future studies.

Type I interferons (IFNs), like IFN-β, strongly inhibit HSV in culture [51] and *C*. *muridarum* infection is known to elicit IFN-β secretion in the murine genital tract [52]. Although *C*. *muridarum* stimulates the production of IFN-β through TLR3 in cell culture [53], TLR3 activation by chlamydiae has not been investigated *in vivo* [53]. TLR3 is activated through binding of dsRNA, a common viral PAMP [54, 55]. Polyinosine-poly(C) (PIC), a synthetic dsRNA analogue, activates TLR3 and protects mice against HSV-2 vaginal challenge when administered prior to HSV-2 infection. PIC-induced protection also coincided with significantly increased detection of IFN-β in vaginal washes [33]. In contrast, genital infection of type I interferon receptor knockout (IFNR-/-) mice with *C*. *muridarum* resulted in reduced bacterial burden compared to wild type mice, indicating that IFN-β production benefits the bacterium rather than the host [56]. The authors postulate that the chlamydiae may stimulate production of IFN-β to inhibit possible viral co-infections and ensure survival in the host genital tract [56]–a prediction that is consistent with our observations. Taken together, published data suggest that *C*. *muridarum*-driven IFN-β production, either through TLR3 or some other pathway, may contribute, at least in part, to the protective effect we observed in our model.

There are also other host responses that may contribute to *Chlamydia*-induced protection, including induction of pro-inflammatory cytokines and/or antimicrobial peptides, stimulation of additional PRRs, or activation of natural killer cells [29, 57–59]. Alternatively, chlamydia-derived products or effectors could directly interfere with viral infection of epithelial cells. For example, the chlamydial protease-like activity factor (CPAF) degrades nectin-1 *in vitro* [60] and may be released into the extracellular environment at the end of the developmental cycle, as proposed by Tang *et al* [61]. Because nectin-1 is the primary co-receptor for HSV-2 in the genital tract [62, 63], CPAF released from chlamydia-infected cells could interfere with HSV-2 infection by reducing host cell surface nectin-1. Though possible, the latter mechanism seems less likely given that HSV infects and replicates with undiminished efficiency within *Chlamydia* pre-infected genital epithelial cells in culture [25]. However, we plan to evaluate each of these possible mechanisms in future studies.

Through the use of a novel *C*. *muridarum* and HSV-2 murine super-infection model, we have described a genital co-infection that benefits the host. As our data demonstrate that chlamydial pre-infection can protect from subsequent HSV-2 challenge, future studies will be focused on dissecting the mechanisms involved in *Chlamydia*-elicited protection. Perhaps more importantly, we wish to understand whether or not chlamydiae specifically activate host defenses that either prevent or enhance establishment of genital infections by other competing pathogenic microorganisms.

## Materials and Methods

### Ethics statement

All animal experiments in this study were conducted in strict accordance with the National Institutes of Health “Guide for the Care and Use of Laboratory Animals”. The animal protocol (110602) was approved by the University Committee on Animal Care at East Tennessee State University under the guidelines of the Association for Assessment and Accreditation of Laboratory Animal Care, US Department of Agriculture, and in compliance with the Public Health Service Policy on Human Care and Use of Laboratory Animals.

### Cells, bacteria and viruses

Cell lines used in the study are HeLa 229 cells, a cervical adenocarcinoma epithelial cell line (ATCC No. CCL2.1) and Vero cells, an African green monkey kidney cell line (ATCC No. CCL-81). Wild type HSV-2 333 strain was used for mouse infections. *C*. *muridarum* Wiess strain was obtained from Kyle Ramsey (Midwestern University). UV-irradiated *C*. *muridarum* was generated as previously described [64]. Briefly, 100–200 μl of *C*. *muridarum* stock was aliquoted into a 24-well plate and placed on a heat sink inside a UV-crosslinker (Spectrolinker XL-1500, Spectronics Corporation) until a minimum dose of 1.0 J cm^-2^ was obtained. Inactivation was confirmed by chlamydial titer assay.

### Animal handling and infections

All mice were provided food and water ad libitum and kept on a standard 12-hour light/dark cycle. After a 1 week acclimation period, 8 week old female BALB/c mice (Harlan, USA) were treated with 2.5 mg Depo-Provera (Greenstone LLC, Peapack, NJ) by subcutaneous injection. Mice were vaginally infected at 9 weeks of age with 10^6^ inclusion forming units (IFU) *C*. *muridarum* on day 0 and then super-infected with either 5 x 10^3^ or 1 x 10^5^ PFU HSV-2 on day 3, 9, or 27 post chlamydial infection (pci) as indicated. All inocula were administered in 10 μl DMEM containing 10% FBS using a micropipette. Mice infected with HSV-2 on day 27 pci were treated with a second dose of Depo-Provera 7 days prior to HSV challenge. Singly-infected, time matched mice were used as controls. In other experiments, mice were infected on day 0 with 10^6^ IFU UV-irradiated, replication-incompetent *C*. *muridarum* then super-infected with HSV-2 on day 3 pci or were infected with *C*. *muridarum* on day 0, treated once with 200 mg/kg Azithromycin (AZM) via oral gavage on day 6 pci and then super-infected with HSV-2 on day 9 pci. Untreated controls were gavaged with sterile water. Finally, in one set of experiments, mice were simultaneously inoculated with 10^6^ IFU of Cm and 5 x 10^3^ PFU of HSV-2. Mock-infected animals were included in each experiment and received 10 μl of media alone on each infection day. All mock-infected animals survived the duration of each experiment and none shed detectable Chlamydia or HSV-2. At the conclusion of each study, or when they exhibited HSV-2-related morbidity, mice were euthanized via cervical dislocation.

To determine pathogen shedding, vaginal swabbing was performed every 3 days, as described, [30]. Preliminary data indicate that swabbing more frequently reduces chlamydial titers and could therefore interfere with the protective effect observed in our model. Swabs were snap frozen in 2 mL tubes containing 500ul DMEM with 10% FBS and three 3mm glass beads and stored at -80°C.

### Monitoring of HSV-2-induced mortality and morbidity

Mice were monitored daily for death or signs of HSV-2 neurologic disease evidenced by unilateral or bilateral hind limb paralysis. External HSV lesions were observed in mice that shed detectable HSV-2, but were not quantified in this study. Mice exhibiting morbidity were sacrificed and incorporated into the survival data as “non-survivors”.

### Chlamydial titer assay

Swab samples were processed as previously described [30]. Briefly, samples were thawed, vortexed and sonicated prior to being diluted for infection of HeLa 229 cells plated at 10^5^ cells per well on glass coverslips in duplicate wells of 24-well plates. Cultures were spin infected for 1 h and refed with antibiotic/antifungal medium. After a 24 h incubation at 35°C, cells were formaldehyde fixed and permeabilized. Chlamydial inclusions were stained with Pathfinder anti-chlamydial LPS fluorescent stain (Bio-rad Laboratories, Hercules, CA) and total number of inclusions per coverslip was recorded. Infectious shedding was reported as average IFU/mouse +/- SEM.

### Plaque assay

Viral recovery from the genital tract was quantified by plaque assay using the same swab sample used to determine chlamydial shedding [31]. Briefly, dilutions ranging from 1:100 to 1:10 were used to infect Vero cell monolayers plated at 1.1 x 10^5^ cells/mL (2 mL/well) in triplicate wells of 6-well plates. Infections were incubated for 1 h at 35°C then inocula were removed and cell layers were overlaid with methylcellulose. After a 72 h incubation at 35°C, the methylcellulose was removed, cell layers were fixed and plaques visualized using crystal violet/formaldehyde stain. Plaques were counted and reported as the average PFU/mouse +/- SEM.

### Statistical analysis

Group survival trends were compared using the log-rank test and were displayed using Kaplan-Meier plots generated by Minitab 16 statistical software. Chlamydial and HSV-2 shedding/recovery between groups was analyzed using Student’s paired t-test. Values of p≤0.05 were considered significant.

## Supporting Information

S1 Fig Simultaneous infection of BALB/c mice with *C*. *muridarum* and HSV-2. Mice were vaginally infected simultaneously with 10 μL of a combined inoculum of 5 x 10^3^ PFU HSV-2 and 10^6^ IFU Cm on day 0 (Sim). Vaginal swabbing was performed every 3 days until day 21 post infection (pi). (A) Morbidity and mortality resulting from HSV-2 was monitored daily until day 21 pi and the percent survival between simultaneously-infected mice and HSV-2 singly-infected controls was compared using the log rank statistic. Significant (p<0.05) differences from the HSV-2 controls are indicated by asterisks (*). The survival curve depicts data from 1 experiment with n = 16 for HSV-2, n = 8 for Cm and n = 12 for Sim-infected group. (B) Viral recovery was determined by plaque assay and is reported as average PFU/mouse +/- SEM. (C) Average HSV-2 recovery at day 3 pi (indicated by bars) and individual mouse HSV-2 recovery (segregated according to survival status) are shown; n = 16 for both HSV-2 (circles) and Cm-3D-H (triangles). Survivors and non-survivors are indicated by S and NS, respectively. Differences in viral recovery between groups were determined with the paired Student’s t-test with p<0.05 considered significant, as indicated by an asterisk (*).
(DOCX)

S2 Fig Super-infection of BALB/c mice with HSV-2 followed by *C*. *muridarum*. (A) Mice were vaginally infected with either 5 x 10^3^ PFU HSV-2 on day 0 post HSV-2 infection (phi), 10^6^ IFU Cm on day 3 phi or super-infected with HSV-2 on day 0 followed by Cm on day 3 phi (HD0, 3D-Cm and H-3D-Cm, respectively). Vaginal swabbing was performed every 3 days until day 21 phi. (B) Morbidity and mortality resulting from HSV-2 was monitored daily until day 24 phi and the percent survival between experimental groups was compared using the log rank statistic. Significant (p<0.05) differences from the HD0 and 3D-Cm controls are indicated by asterisks (*). The survival curve depicts data from 1 experiment with n = 18 for the HD0 and H-3D-Cm groups and n = 8 for the 3D-Cm group. (C) Chlamydial shedding was determined by chlamydial titer assay and is reported as average IFU/mouse +/- SEM. (D) Average chlamydial shedding at day 6 phi (indicated by bars) and individual mouse chlamydial shedding (segregated according to survival status) are shown; n = 8 for 3D-Cm (circles), and n = 18 for H-3D-Cm (triangles). (E) HSV-2 recovery was determined by plaque assay and is reported as average PFU/mouse +/- SEM. (F) Average HSV-2 recovery at day 3 phi (indicated by bars) and individual mouse HSV-2 recovery (segregated according to survival status) are shown; n = 18 for both HSV-2 (circles) and UVCm-3D-H (triangles). Survivors and non-survivors are indicated by S and NS, respectively. Differences in pathogen shedding/recovery between groups were determined with the paired Student’s t-test with p<0.05 considered significant, as indicated by an asterisk (*).
(DOCX)
