# Supplementary material for: Chlamydial Pre-Infection Protects from Subsequent Herpes Simplex Virus-2 Challenge in a Murine Vaginal Super-Infection Model
Source: PLoS One. 2016 Jan 4;11(1):e0146186. doi: 10.1371/journal.pone.0146186 (PMC4699815; doi:10.1371/journal.pone.0146186)
Supplement: S1 Fig — Mice were vaginally infected simultaneously with 10 μL of a combined inoculum of 5 x 10^3^ PFU HSV-2 and 106 IFU Cm on day 0 (Sim). Vaginal swabbing was performed every 3 days until day 21 post infection (pi). (A) Morbidity and mortality resulting from HSV-2 was monitored daily until day 21 pi and the percent survival between simultaneously-infected mice and HSV-2 singly-infected controls was compared using the log rank statistic. Significant (p<0.05) differences from the HSV-2 controls are indicated by asterisks (*). The survival curve depicts data from 1 experiment with n = 16 for HSV-2, n = 8 for Cm and n = 12 for Sim-infected group. (B) Viral recovery was determined by plaque assay and is reported as average PFU/mouse +/- SEM. (C) Average HSV-2 recovery at day 3 pi (indicated by bars) and individual mouse HSV-2 recovery (segregated according to survival status) are shown; n = 16 for both HSV-2 (circles) and Cm-3D-H (triangles). Survivors and non-survivors are indicated by S and NS, respectively. Differences in viral recovery between groups were determined with the paired Student’s t-test with p<0.05 considered significant, as indicated by an asterisk (*). (DOCX) [file pone.0146186.s001.docx]

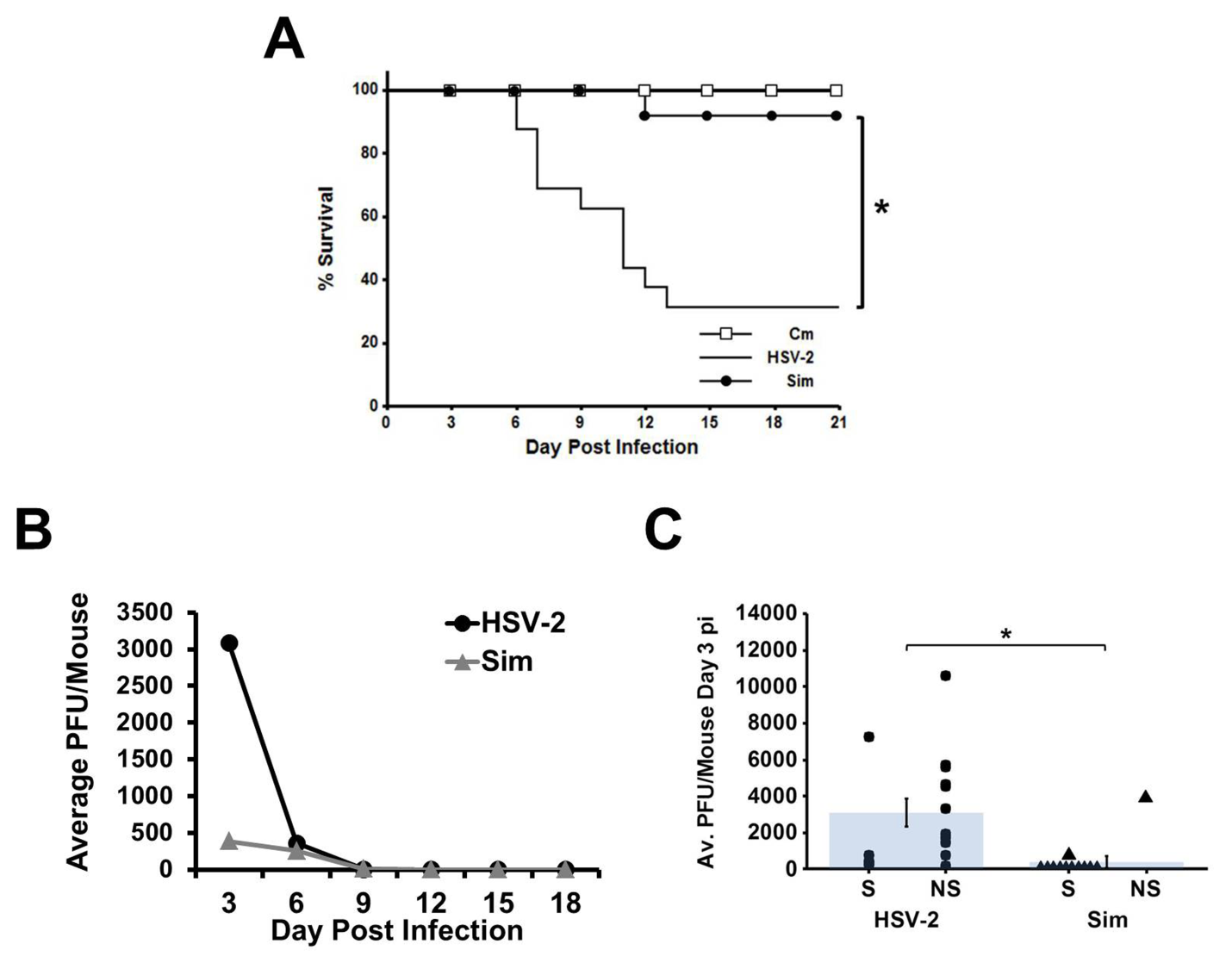


**Figure S1. Simultaneous infection of BALB/c mice with *C. muridarum* and HSV-2.**  Mice were vaginally infected simultaneously with 10 µL of a combined inoculum of 5 x 10^3^ PFU HSV-2 and 10^6^ IFU Cm on day 0 (Sim). Vaginal swabbing was performed every 3 days until day 21 post infection (pi). (A) Morbidity and mortality resulting from HSV-2 was monitored daily until day 21 pi and the percent survival between simultaneously-infected mice and HSV-2 singly-infected controls was compared using the log rank statistic. Significant (p<0.05) differences from the HSV-2 controls are indicated by asterisks (*). The survival curve depicts data from 1 experiment with n=16 for HSV-2, n=8 for Cm and n=12 for Sim-infected group. (B) Viral shedding was determined by plaque assay and is reported as average PFU/mouse +/- SEM. (C) Average HSV-2 shedding at day 3 pi (indicated by bars) and individual mouse HSV-2 shedding (segregated according to survival status) are shown; n=16 for both HSV-2 (circles) and Cm-3D-H (triangles). Survivors and non-survivors are indicated by S and NS, respectively. Differences in viral shedding between groups were determined with the paired Student’s t-test with p<0.05 considered significant, as indicated by an asterisk (*).
