# Supplementary material for: Chlamydial Pre-Infection Protects from Subsequent Herpes Simplex Virus-2 Challenge in a Murine Vaginal Super-Infection Model
Source: PLoS One. 2016 Jan 4;11(1):e0146186. doi: 10.1371/journal.pone.0146186 (PMC4699815; doi:10.1371/journal.pone.0146186)
Supplement: S2 Fig — (A) Mice were vaginally infected with either 5 x 10^3^ PFU HSV-2 on day 0 post HSV-2 infection (phi), 106 IFU Cm on day 3 phi or super-infected with HSV-2 on day 0 followed by Cm on day 3 phi (HD0, 3D-Cm and H-3D-Cm, respectively). Vaginal swabbing was performed every 3 days until day 21 phi. (B) Morbidity and mortality resulting from HSV-2 was monitored daily until day 24 phi and the percent survival between experimental groups was compared using the log rank statistic. Significant (p<0.05) differences from the HD0 and 3D-Cm controls are indicated by asterisks (*). The survival curve depicts data from 1 experiment with n = 18 for the HD0 and H-3D-Cm groups and n = 8 for the 3D-Cm group. (C) Chlamydial shedding was determined by chlamydial titer assay and is reported as average IFU/mouse +/- SEM. (D) Average chlamydial shedding at day 6 phi (indicated by bars) and individual mouse chlamydial shedding (segregated according to survival status) are shown; n = 8 for 3D-Cm (circles), and n = 18 for H-3D-Cm (triangles). (E) HSV-2 recovery was determined by plaque assay and is reported as average PFU/mouse +/- SEM. (F) Average HSV-2 recovery at day 3 phi (indicated by bars) and individual mouse HSV-2 recovery (segregated according to survival status) are shown; n = 18 for both HSV-2 (circles) and UVCm-3D-H (triangles). Survivors and non-survivors are indicated by S and NS, respectively. Differences in pathogen shedding/recovery between groups were determined with the paired Student’s t-test with p<0.05 considered significant, as indicated by an asterisk (*). (DOCX) [file pone.0146186.s002.docx]

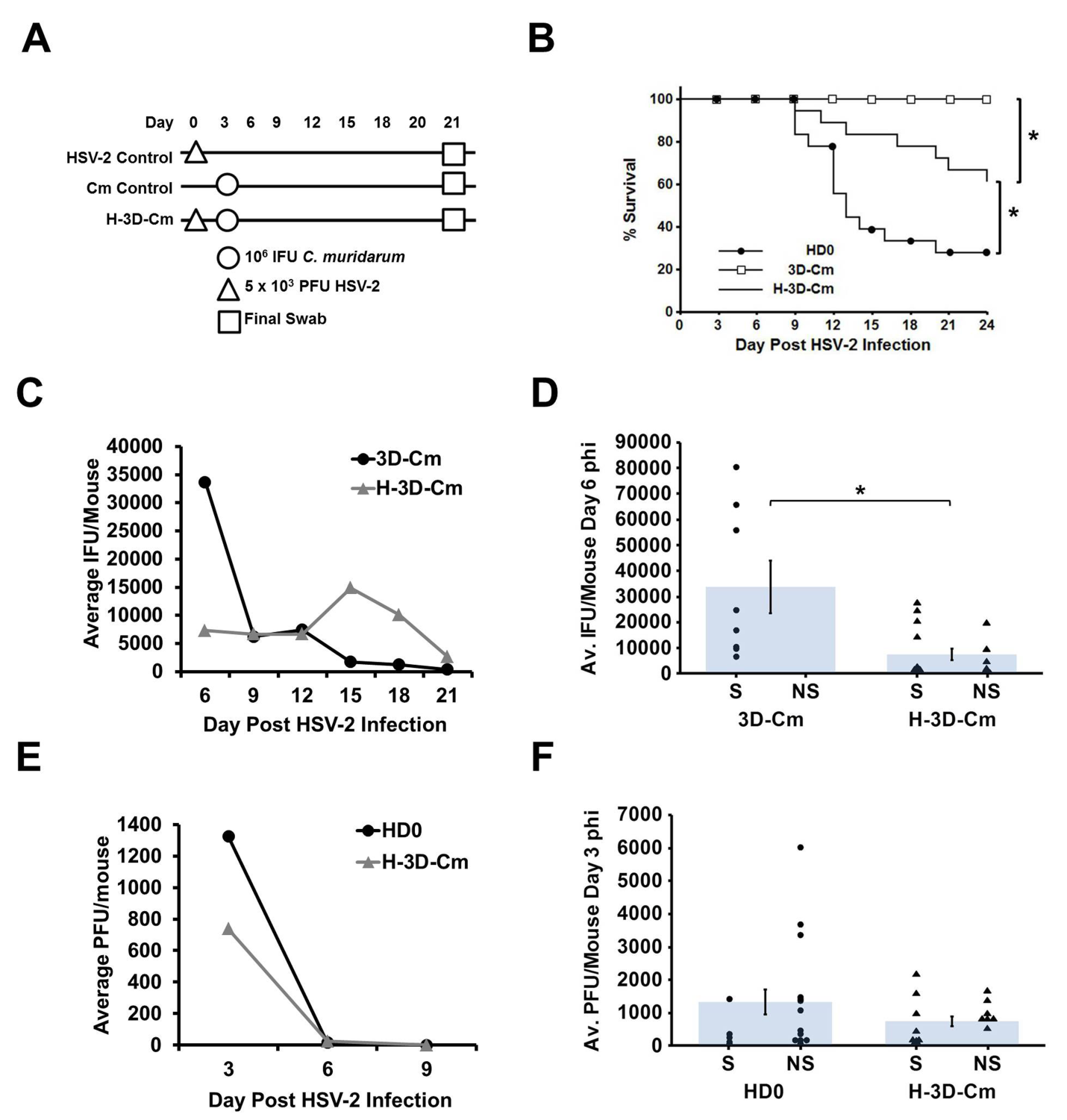


**Figure S2. Super-infection of BALB/c mice with HSV-2 followed by *C. muridarum*.** (A) Mice were vaginally infected with either 5 x 10^3^ PFU HSV-2 on day 0 post HSV-2 infection (phi), 10^6^ IFU Cm on day 3 phi or super-infected with HSV-2 on day 0 followed by Cm on day 3 phi (HD0, 3D-Cm and H-3D-Cm, respectively). Vaginal swabbing was performed every 3 days until day 21 phi. (B) Morbidity and mortality resulting from HSV-2 was monitored daily until day 24 phi and the percent survival between experimental groups was compared using the log rank statistic. Significant (p<0.05) differences from the HD0 and 3D-Cm controls are indicated by asterisks (*). The survival curve depicts data from 1 experiment with n=18 for the HD0 and H-3D-Cm groups and n=8 for the 3D-Cm group. (C) Chlamydial shedding was determined by chlamydial titer assay and is reported as average IFU/mouse +/- SEM. (D) Average chlamydial shedding at day 6 phi (indicated by bars) and individual mouse chlamydial shedding (segregated according to survival status) are shown; n=8 for 3D-Cm (circles), and n=18 for H-3D-Cm (triangles). (E) HSV-2 shedding was determined by plaque assay and is reported as average PFU/mouse +/- SEM. (F) Average HSV-2 shedding at day 3 phi (indicated by bars) and individual mouse HSV-2 shedding (segregated according to survival status) are shown; n=18 for both HSV-2 (circles) and UVCm-3D-H (triangles). Survivors and non-survivors are indicated by S and NS, respectively. Differences in pathogen shedding between groups were determined with the paired Student’s t-test with p<0.05 considered significant, as indicated by an asterisk (*).
